# Unraveling the metabolic pathways between atherosclerosis and sarcopenia

**DOI:** 10.3389/fendo.2025.1762825

**Published:** 2026-02-06

**Authors:** Mei Yu, Lichao Ge, Chen Fu, Rujia Zhao

**Affiliations:** The First People’s Hospital of Xiaoshan District, Xiaoshan Affiliated Hospital of Wenzhou Medical University, Hangzhou, China

**Keywords:** aging, atherosclerosis, inflammaging, insulin resistance, sarcopenia

## Abstract

Sarcopenia and atherosclerosis are age-related conditions pathologically intertwined through a self-reinforcing, bidirectional cycle. This review dissects the core mechanistic pillars of this synergy such as insulin resistance, chronic low-grade inflammation, ectopic lipid deposition, and hormonal dysregulation. We detail how skeletal muscle dysfunction exacerbates systemic insulin resistance and inflammatory cascades that accelerate endothelial damage and atherogenesis. Conversely, atherosclerotic vascular impairment compromises microcirculatory function, inducing muscle ischemia and metabolic decline. Beyond pathogenesis, we evaluate integrated intervention, including combined exercise, anti-inflammatory diets, and pleiotropic pharmacotherapies, that concurrently target shared pathways in muscle and vasculature. By framing this comorbidity within the context of aging hallmarks, we advocate a paradigm shift from organ-specific management toward a holistic, geroscience-based approach to mitigate frailty and disability in the aging population.

## 1 Introduction

Sarcopenia and atherosclerosis are major drivers of morbidity and mortality in older adults. Traditionally, they have been studied in isolation, but a burgeoning body of evidence now positions them as manifestations of a shared systemic age-associated metabolic dysfunction (1). The interplay between deteriorating muscle health and progressive vascular disease is mediated by fundamental biological processes of aging, including dysregulated nutrient sensing, mitochondrial dysfunction, and altered intercellular communication (2, 3). Traditionally studied as distinct entities, emerging evidence now reveals a complex bidirectional relationship mediated by shared metabolic pathways, including insulin resistance (IR), chronic inflammation, ectopic fat deposition, and hormonal Shifts (4–7). Epidemiological studies indicate that sarcopenia is associated with an increased risk of cardiovascular diseases (6, 8), including atherosclerosis, independent of traditional risk factors. Conversely, atherosclerosis and its risk factors, such as metabolic syndrome (MetS), type 2 diabetes mellitus (T2DM), and visceral obesity, can accelerate muscle loss, creating a vicious cycle that exacerbates both conditions (9, 10). The interplay between skeletal muscle and vascular health is thus a critical area of research with significant implications for early intervention and holistic management. At the molecular level, IR is a central player. Skeletal muscle is a primary site for insulin-mediated glucose uptake; consequently, muscle atrophy contributes to systemic IR (11), which in turn promotes endothelial dysfunction (12). Ectopic fat deposition, particularly in the liver and muscle, is another hallmark of both conditions, driven by lipid spillover from dysfunctional adipose tissue (13–15). Chronic low-grade inflammation (“inflammaging”), characterized by elevated cytokines such as interleukin-6 (IL-6) and tumor necrosis factor-alpha (TNF-α), further links muscle wasting to vascular damage (16–18). Vitamin D deficiency, common in both disorders, may also serve as a modulatory factor, influencing muscle protein synthesis and vascular inflammation (19, 20). Additionally, myokines and adipokines, such as myostatin, adiponectin, and irisin, form a cross-tissue network that regulates metabolism and inflammation, offering potential therapeutic targets (16–18). This review synthesizes current evidence on the bidirectional metabolic crosstalk between these conditions, adopting a geroscience framework to elucidate how targeting core aging mechanisms may offer synergistic benefits for both muscle and vascular health.

## 2 Sarcopenia and metabolic mechanism

Sarcopenia is a multifactorial syndrome driven by a complex interplay of metabolic dysregulations that create a self-perpetuating cycle of muscle wasting (21). A central mechanism is IR and compensatory hyperinsulinemia. Given that skeletal muscle accounts for approximately 80% of postprandial glucose uptake (22), its quantity and quality are fundamental determinants of systemic glucose homeostasis. Reduced muscle mass directly diminishes glucose disposal capacity, leading to hyperinsulinemia, which exerts direct catabolic effects on muscle (21). This disrupts the phosphoinositide 3-kinase (PI3K)/Akt signaling pathway, crucial for protein synthesis. Impaired insulin signaling activates forkhead box O (FoxO) transcription factors, upregulating atrophy-related genes like Atrogin-1 and MuRF-1, which promote proteasomal degradation of muscle proteins (23). Concurrently, IR increases lipolysis, elevating circulating free fatty acids (FFAs) that accumulate intramyocellularly lipids (IMCL), particularly diacylglycerols (DAGs) and ceramides (24). These lipid intermediates activate inflammatory pathways like nuclear factor-κB (NF-κB) and directly inhibit insulin signaling, further exacerbating IR (24). Beyond proteolysis, IR and aging contribute to anabolic resistance, whereby muscle becomes less responsive to the protein-synthesis-stimulating effects of both insulin and amino acids, further hindering maintenance and repair (25).

This metabolic dysfunction is exacerbated by inflammaging, characterized by elevated pro-inflammatory cytokines such as TNF-α, IL-6, and C-reactive protein (CRP) (26). TNF-α is a potent inducer of muscle wasting, activating NF-κB to stimulate MuRF-1 expression and protein breakdown (23). IL-6 can induce atrophy via the Janus kinase-signal transducer and activator of transcription (JAK/STAT) pathway and by suppressing Insulin/insulin-like growth factor-1 (IGF-1) signaling (27). In visceral obesity, dysfunctional adipose tissue infiltrated by macrophages becomes a significant source of these cytokines, creating a systemic catabolic environment for muscle (28).

Mitochondrial dysfunction is another key pillar of sarcopenia. The age-related decline in mitochondrial biogenesis, impaired oxidative phosphorylation (OXPHOS), and increased reactive oxygen species (ROS) production create an energetic deficit, compromising ATP-intensive processes like protein synthesis and sarcomere maintenance (29). Excessive ROS damages cellular components, can trigger apoptosis, and is intrinsically linked to intramuscular IR (30). These processes are compounded by hormonal changes. Vitamin D deficiency, common in aging and MetS, is associated with muscle weakness and atrophy, likely by impairing myocyte differentiation, calcium handling, and inflammation modulation (31). The age-related decline in the Growth Hormone (GH)/IGF-1 axis reduces a vital anabolic stimulus for muscle protein synthesis, as IGF-1 is a primary activator of the Protein Kinase B/Mammalian Target of Rapamycin (Akt/mTOR) pathway (32). The decline in sex hormones, particularly testosterone in men, further reduces anabolic support by directly stimulating synthesis and inhibiting breakdown (33). In addition, sex hormones not only directly regulate muscle anabolism, but also indirectly participate in the common pathological process of sarcopenia and atherosclerosis by influencing fat distribution and metabolic phenotype. Estrogen tends to promote subcutaneous storage of fat and inhibit visceral fat accumulation and inflammation, while androgens can inhibit fat differentiation and promote lipolysis at physiological levels; After menopause, women experience a sudden drop in estrogen levels, leading to a shift in fat distribution from “subcutaneous dominance” to “visceral dominance”, accompanied by increased inflammation of adipose tissue and lipid leakage (34, 35). The expansion of visceral fat not only directly activates the muscle atrophy pathway and inhibits protein synthesis by releasing inflammatory factors such as IL-6 and TNF –α (36), but also drives IR and ectopic lipid deposition in muscles, further damaging muscle mass and function (37). At the same time, FFAs from visceral fat and inflammatory mediators enter the liver through the portal vein to promote the formation of atherogenic lipoprotein profile, and cooperate with the endothelial function decline caused by estrogen loss to jointly accelerate vascular disease (38, 39). Therefore, gender differences in fat distribution regulated by sex hormones are an important link connecting muscle and vascular metabolic dysfunction.

Critically, the interplay between sarcopenia and obesity converges into a distinct clinical phenotype known as sarcopenic obesity, which represents a high-risk geriatric syndrome characterized by the co-existence of reduced muscle mass/function and excess adiposity (10, 40). This condition is not merely the sum of its components but results from a synergistic pathophysiology that accelerates both musculoskeletal and cardiometabolic decline. The core mechanisms driving sarcopenic obesity are the same shared metabolic pathways linking sarcopenia and atherosclerosis: profound IR, chronic inflammation, and ectopic lipid deposition (41, 42). In sarcopenic obesity, the loss of metabolically active muscle mass diminishes glucose disposal and basal metabolic rate, promoting further adiposity and systemic IR. Concurrently, hypertrophic and dysfunctional adipose tissue, particularly visceral fat, releases elevated levels of FFAs and pro-inflammatory cytokines that promote muscle protein breakdown via ubiquitin-proteasome activation, inhibit anabolic signaling, and induce intramyocellular lipid accumulation, thereby creating a self-perpetuating cycle of muscle loss and fat gain (43, 44).

**Figure 1:**
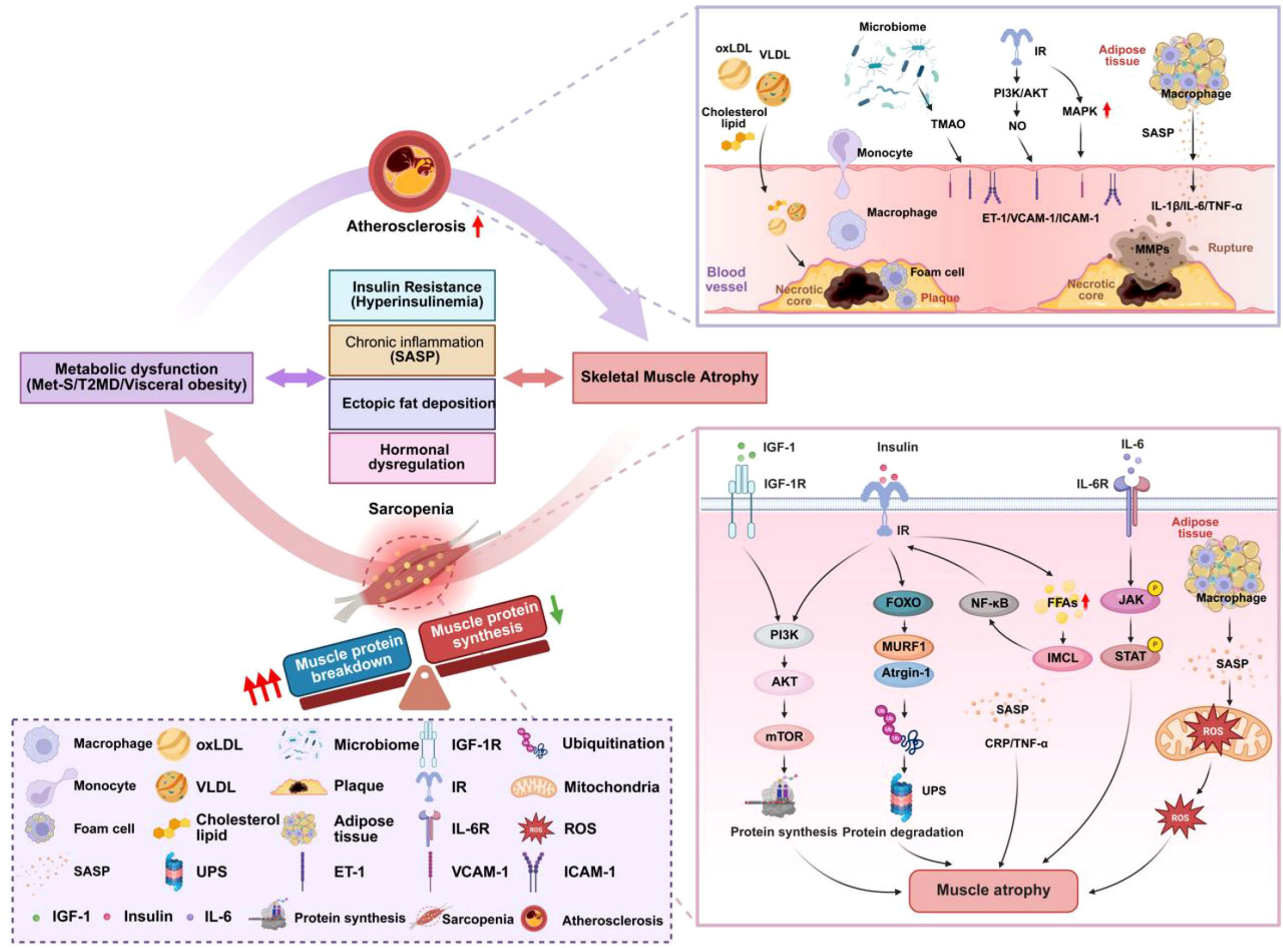
Schematic overview of the core metabolic pathways linking sarcopenia and atherosclerosis. This figure illustrates the four key pathophysiological pillars that create a vicious bidirectional cycle between muscle and vascular decline: (1) Systemic Insulin Resistance drives muscle atrophy and impairs vascular endothelial function. (2) Inflammaging, fueled by visceral fat and cellular senescence, simultaneously promotes muscle protein breakdown and atherosclerotic plaque progression. (3) Ectopic Lipid Deposition from adipose tissue dysfunction leads to intramyocellular lipotoxicity and promotes atherogenic dyslipidemia. (4) Hormonal Dysregulation creates a shared catabolic and pro-inflammatory state. Arrows indicate the bidirectional crosstalk and positive feedback loops that perpetuate the co-development of both conditions.

Finally, ectopic fat deposition results from the inability of subcutaneous fat to expand healthily, leading to lipid spillover into muscle (45). IMCL and their derivatives (ceramides, DAGs) actively disrupt insulin signaling and promote inflammation (24). This infiltration is a critical determinant of muscle quality; individuals with identical muscle mass can have vastly different strength and metabolic profiles based on their degree of fatty infiltration, which weakens muscle architecture and contractile force (46) (As shown in **Figure 1**).

## 3 Atherosclerosis and metabolic mechanism

Atherosclerosis is a chronic inflammatory disease of the arterial wall, whose pathogenesis is deeply intertwined with systemic metabolic dysfunction (47). The initial insult often stems from endothelial dysfunction. Under physiological conditions, insulin promotes vasodilation by activating the PI3K/Akt pathway to stimulate endothelial nitric oxide synthase (eNOS) and increase bioavailable nitric oxide (NO) (48). In the insulin-resistant state, this pathway is selectively impaired, reducing NO bioavailability. Concurrently, other insulin signaling pathways (e.g., MAPK) remain active, driving a pathogenic shift towards increased secretion of the vasoconstrictor endothelin-1 (ET-1), upregulation of adhesion molecules (VCAM-1, ICAM-1), and elevated expression of plasminogen activator inhibitor-1 (PAI-1), fostering a pro-inflammatory, pro-thrombotic milieu that initiates atherogenesis (48).

This endothelial dysfunction is fueled by atherogenic dyslipidemia. IR drives hepatic overproduction of large, triglyceride-rich very-low-density lipoproteins (VLDL) (49). Elevated triglycerides facilitate a cholesteryl ester transfer protein (CETP)-mediated exchange, remodeling LDL into small, dense LDL (sdLDL) particles, which are highly atherogenic due to increased susceptibility to oxidation and enhanced arterial retention (49). Concomitantly, high-density lipoproteins (HDL) become triglyceride-enriched and cholesterol-depleted, transforming it from a protective particle into a dysfunctional or pro-inflammatory state (50).

The retention and oxidation of low-density lipoproteins (LDL) within the subendothelial space forms Oxidized LDL (oxLDL), a pivotal “danger signal” that triggers a robust inflammatory response (47). OxLDL activates the endothelium, promoting monocyte recruitment and differentiation into macrophages. These macrophages engulf modified lipoproteins via scavenger receptors, becoming lipid-laden “foam cells” that define the early fatty streak lesion (51). These activated immune cells secrete pro-inflammatory cytokines (e.g., IL-1β, IL-6, TNF-α), chemokines, and growth factors that perpetuate leukocyte recruitment and drive plaque progression. Inflammation also dictates clinical outcomes; matrix metalloproteinases (MMPs) secreted by macrophages degrade the plaque’s fibrous cap, rendering it vulnerable to rupture and precipitating acute thrombotic events like myocardial infarction or stroke (47, 51).

The systemic inflammatory tone is powerfully modulated by visceral adipose tissue (VAT). In obesity, hypertrophied adipocytes and infiltrating immune cells within VAT secrete elevated levels of pro-inflammatory adipokines while suppressing protective ones like adiponectin (52, 53). Via drainage into the portal circulation, VAT floods the liver with FFAs and inflammatory mediators, exacerbating hepatic IR, promoting dyslipidemia, and stimulating the production of acute-phase proteins like CRP, thereby amplifying the systemic inflammatory burden (38, 54).

Emerging research also implicates the gut microbiome. Dietary nutrients rich in choline and L-carnitine are metabolized by gut microbes into trimethylamine, which is oxidized in the liver to trimethylamine N-oxide (TMAO). Elevated TMAO levels are associated with increased cardiovascular risk, as it promotes atherosclerosis by enhancing foam cell formation, activating inflammatory pathways, and impairing endothelial function (55) (As shown in **Figure 1**).

## 4 Shared metabolic pathways in sarcopenia and atherosclerosis

The pathophysiological convergence of sarcopenia and atherosclerosis is not merely associative but causal, creating a feed-forward loop of decline. This cycle is powered by the dysfunction of evolutionarily conserved metabolic and inflammatory pathways, which are also core pillars of the aging process itself (As shown in **Table 1**).

**Table 1:** Bidirectional metabolic mechanisms linking sarcopenia and atherosclerosis.

Shared metabolic pathway	Sarcopenia	Atherosclerosis	Bidirectional vicious cycle
Insulin resistance	• Impairs PI3K/Akt signaling, leading to reduced protein synthesis. • Activates FoxO transcription factors, upregulating Atrogin-1 and MuRF-1 → proteasomal degradation. • Induces anabolic resistance to amino acids. • Promotes IMCLs accumulation (DAGs, ceramides) → exacerbates IR.	• Selective impairment of PI3K/Akt/eNOS pathway → reduced NO bioavailability. • Unchecked MAPK signaling → increased endothelin-1, adhesion molecules (VCAM-1, ICAM-1), PAI-1. • Promotes endothelial dysfunction, inflammation, and thrombosis.	• Muscle IR → systemic hyperinsulinemia → endothelial dysfunction. • Vascular IR → reduced blood flow → muscle ischemia → worsens sarcopenia.
Inflammaging	• TNF-α activates NF-κB → upregulates MuRF-1/Atrogin-1 → muscle breakdown. • IL-6 via JAK/STAT → suppresses IGF-1/Akt/mTOR → inhibits protein synthesis. • Macrophage infiltration → cytokine release (TNF-α, IL-1β) → impairs satellite cell function.	• Cytokines activate endothelium → adhesion molecule expression → monocyte recruitment. • oxLDL uptake → foam cell formation. • MMPs secretion → plaque destabilization. •→ systemic cytokine release.	• VAT-derived cytokines (e.g., IL-6, TNF-α) simultaneously damage muscle and vasculature. • Systemic inflammation → mutual amplification of tissue degradation.
Lipid Dysregulation & Ectopic Fat Deposition	• IR → increased lipolysis → elevated FFAs →IMCLs • DAGs and ceramides inhibit insulin signaling → local IR. • Ceramides activate PP2A → dephosphorylates Akt → promotes atrophy.	• Hepatic VLDL overproduction → CETP-mediated lipid exchange →sdLDL and dysfunctional HDL. • sdLDL → increased oxidation, arterial retention → foam cells. • Dysfunctional HDL → loss of reverse cholesterol transport.	• Ectopic fat in muscle worsens IR → promotes atherogenic dyslipidemia. • Dyslipidemia → systemic inflammation → exacerbates muscle catabolism.
Hormonal Dysregulation	• Vitamin D deficiency: impairs myogenesis, calcium handling, and regeneration. • Sex hormone decline: reduced testosterone/estrogen → decreased anabolic signaling via Akt/mTOR. • GH/IGF-1 decline: loss of anabolic stimulus → impaired protein synthesis.	• Vitamin D deficiency: increases ROS, reduces NO, upregulates NF-κB → endothelial dysfunction. • Sex hormone decline: loss of eNOS stimulation → vasoconstriction, inflammation. • IGF-1 decline: endothelial dysfunction, vascular stiffness.	• Hormonal deficits create a catabolic milieu affecting both muscle and vasculature. • Low vitamin D → concurrent muscle atrophy and vascular inflammation.

### 4.1 Insulin resistance

IR represents a fundamental and shared metabolic defect that fuels a self-perpetuating, bidirectional pathological cycle between skeletal muscle and the vasculature, thereby accelerating the progression of both sarcopenia and atherosclerosis (56, 57). Skeletal muscle, being the primary site for postprandial glucose disposal, sees its metabolic function critically impaired in sarcopenia. The reduction in muscle mass and quality directly diminishes the body’s capacity for insulin-mediated glucose clearance, leading to compensatory hyperinsulinemia (21, 58). This hyperinsulinemia is not merely a marker of compensation but an active contributor to pathology, as it exerts direct catabolic effects on muscle tissue and promotes endothelial dysfunction (58). At the molecular level within myocytes, IR disrupts the anabolic PI3K/Akt signaling pathway. This impairment not only blunts protein synthesis but also leads to the activation of FoxO transcription factors. Activated FoxO upregulates the expression of the muscle-specific E3 ubiquitin ligases Atrogin-1 and MuRF-1, orchestrating the proteasomal degradation of key contractile proteins and driving muscle atrophy (59). Concurrently, IR induces a state of anabolic resistance, whereby the skeletal muscle becomes less responsive to the protein-synthetic stimulus of both insulin and essential amino acids, further crippling its maintenance and repair capabilities (60, 61). The metabolic consequences extend beyond glucose, as IR in adipose tissue triggers enhanced lipolysis, elevating circulating FFAs. These FFAs are taken up by muscle and esterified into toxic lipid intermediates like DAGs and ceramides. DAGs activate protein kinase C (PKC) isoforms that serine-phosphorylate and inhibit the insulin receptor substrate 1 (IRS-1), while ceramides activate Protein Phosphatase 2A (PP2A), which dephosphorylates and deactivates Akt, thereby locally exacerbating IR and promoting further atrophy (56, 62).

**Figure 2:**
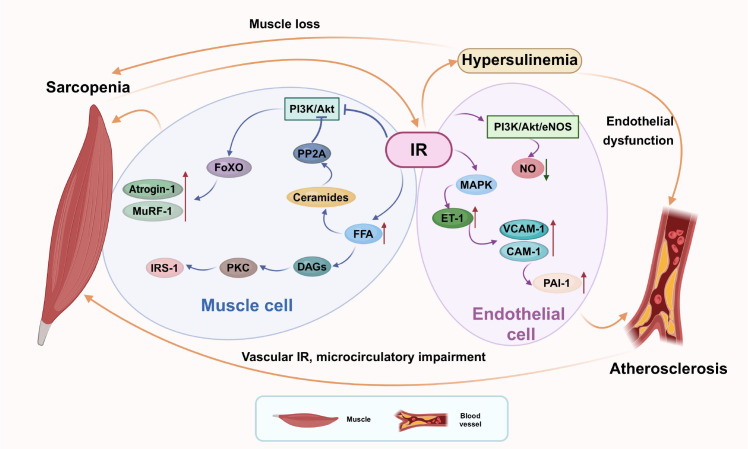
Insulin resistance as a bidirectional driver of sarcopenia and atherosclerosis. Skeletal muscle insulin resistance impairs glucose uptake and promotes atrophy, exacerbating systemic insulin resistance and hyperinsulinemia. Concurrently, vascular insulin resistance selectively impairs the PI3K/Akt/eNOS pathway, reducing NO bioavailability while promoting pro-inflammatory MAPK signaling. This results in endothelial dysfunction, reduced muscle perfusion, and a feed-forward cycle that worsens both conditions.

Conversely, the impact of IR on the vascular endothelium is a primary driver of atherogenesis and directly compromises muscle health. In a state of IR, insulin signaling in endothelial cells becomes selectively impaired in the PI3K/Akt/eNOS axis. This results in reduced production of the vasoprotective molecule NO (63, 64). Critically, the MAPK pathway remains sensitized to insulin, leading to a pathological imbalance. This unchecked MAPK signaling promotes the overexpression of the potent vasoconstrictor ET-1, upregulates the expression of adhesion molecules such as VCAM-1 and ICAM-1, and increases the secretion of PAI-1. This shift creates a pro-inflammatory, pro-thrombotic, and pro-atherogenic endothelial phenotype that initiates and accelerates plaque formation (48, 65). The resulting endothelial dysfunction and the associated microvascular rarefaction significantly impair blood flow and nutrient delivery to skeletal muscle. This creates a state of relative muscle ischemia, which exacerbates metabolic stress, limits exercise capacity, and contributes to further muscle wasting, thereby directly feeding into the sarcopenic process (5, 57, 63) (As shown in **Figure 2**).

This intricate crosstalk establishes a feed-forward vicious cycle: sarcopenia-induced systemic IR worsens endothelial health, while vascular IR and microcirculatory impairment hinder muscle perfusion and metabolism, deepening sarcopenia.

### 4.2 Inflammaging

Chronic, low-grade inflammation, termed “inflammaging,” is a cornerstone of the aging process and a critical bidirectional link between sarcopenia and atherosclerosis. This persistent inflammatory state is not merely a passive association but an active driver of pathology in both muscle and vasculature, creating a self-reinforcing cycle of tissue degeneration (66, 67). The expansion and dysfunction of VAT serve as a primary hub for systemic inflammaging (68). In obesity and aging, hypertrophied adipocytes and infiltrating immune cells, particularly pro-inflammatory M1 macrophages, secrete a plethora of inflammatory mediators, including TNF-α, IL-6, and IL-1β (69, 70). This VAT-derived cytokine flood, drained into the portal circulation, perpetuates a state of chronic systemic inflammation that simultaneously attacks skeletal muscle and the arterial wall (71–74).

In skeletal muscle, these circulating cytokines activate distinct pro-atrophic pathways. TNF-α robustly activates the IκB kinase/NF-κB signaling cascade. NF-κB translocation to the nucleus directly transcribes the genes encoding MuRF-1, thereby accelerating the ubiquitin-proteasome system-mediated breakdown of myofibrillar proteins (75). IL-6, in a dualistic manner, can signal through its membrane-bound receptor or via a soluble receptor (trans-signaling). Chronic IL-6 exposure, particularly via trans-signaling, activates the JAK/STAT pathway. This leads to the upregulation of Suppressor of Cytokine Signaling 3 (SOCS3), which directly inhibits IGF-1 receptor signaling, thereby blunting the critical PI3K/Akt/mTOR anabolic pathway necessary for muscle protein synthesis and repair (76–78). Furthermore, local inflammation within the muscle milieu, characterized by M1 macrophage infiltration, impairs the function of satellite cells severely compromising the regenerative capacity of skeletal muscle in response to damage or stress (79–81).

In parallel, the same inflammatory mediators potently drive atherosclerotic progression. TNF-α and IL-1β activate the vascular endothelium, increasing the expression of adhesion molecules (e.g., VCAM-1, ICAM-1) and promoting the recruitment of monocytes into the subendothelial space (82). Within the nascent plaque, these monocytes differentiate into macrophages, which engulf oxLDL to become lipid-laden foam cells, the hallmark of early atherosclerotic lesions (83). These activated immune cells further produce additional cytokines (IL-6, TNF-α) and MMPs, the latter of which degrade the fibrous cap of advanced plaques, rendering them vulnerable to rupture and causing acute thrombotic events like myocardial infarction (84).

**Figure 3:**
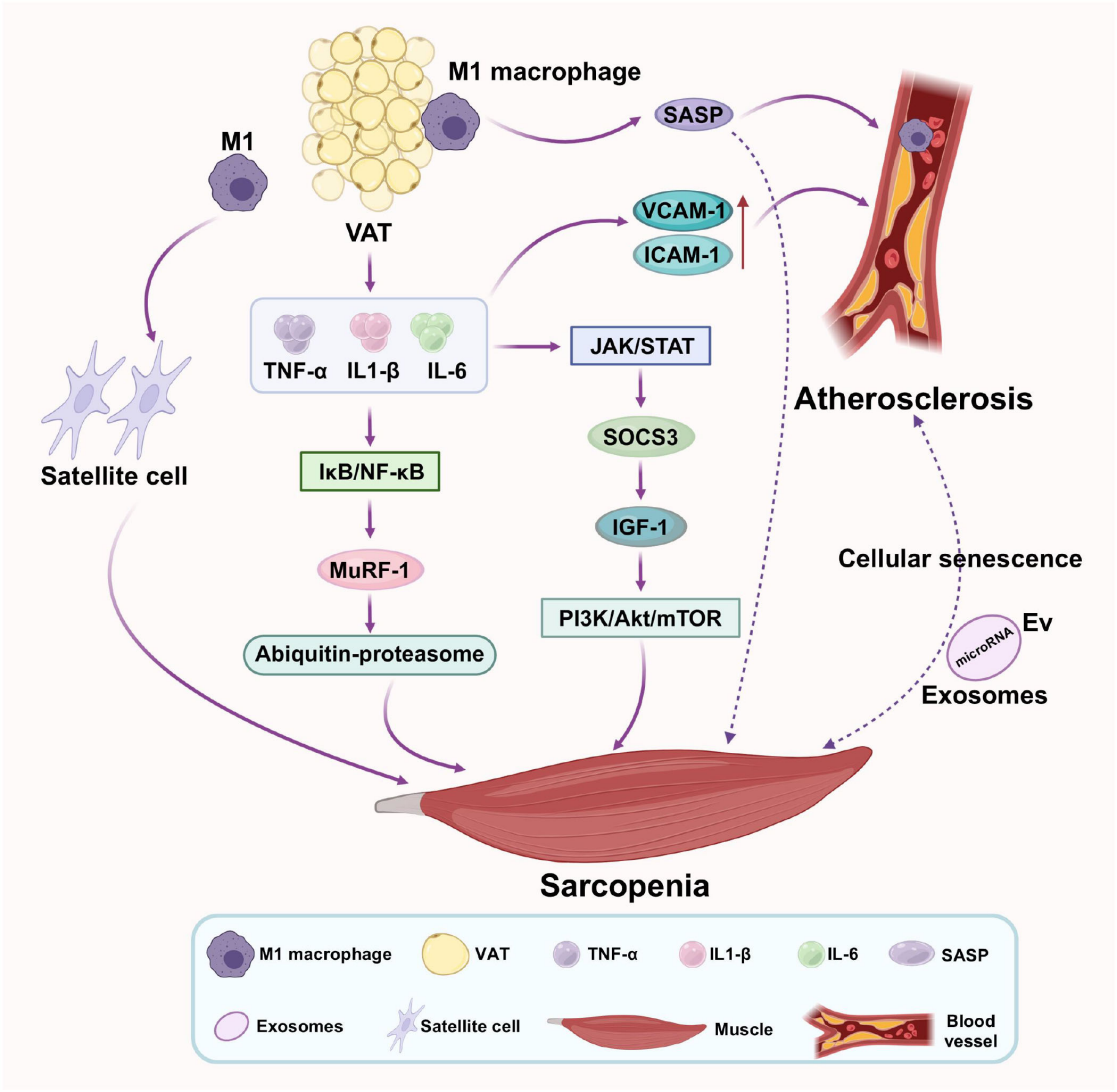
Inflammaging as a critical bidirectional link between sarcopenia and atherosclerosis. Chronic inflammation, driven by visceral adipose tissue and cellular senescence, releases pro-inflammatory cytokines (TNF-α, IL-6, IL-1β). These cytokines simultaneously activate muscle catabolic pathways (NF-κB/JAK-STAT) and vascular inflammatory responses, promoting muscle protein breakdown, endothelial dysfunction, monocyte recruitment, and plaque progression, thereby creating a self-reinforcing inflammatory cycle.

Cellular senescence, a state of irreversible growth arrest, is a key contributor. Senescent cells accumulate with age in both muscle and vasculature and secrete a powerful cocktail of pro-inflammatory factors, proteases, and growth factors known as the senescence-associated secretory phenotype (SASP) (3). The SASP directly promotes muscle fiber atrophy and endothelial dysfunction, creating a locally aggravated inflammatory environment (85, 86). Extracellular vesicles (EVs), including exosomes, have been identified as novel vehicles for inter-tissue communication. For instance, endothelial-derived EVs carrying specific microRNAs (e.g., miR-92a) can be taken up by skeletal muscle cells, where they suppress insulin signaling and promote atrophy. Conversely, EVs from atrophying muscle may carry pro-inflammatory cargo that can activate endothelial cells (87). The gut microbiome also plays a role; dysbiosis can lead to increased intestinal permeability, allowing bacterial lipopolysaccharide to enter the circulation, a condition known as metabolic endotoxemia, which triggers systemic inflammation through Toll-like receptor signaling, impacting both muscle and vasculature (88, 89). In summary, inflammaging is not a background phenomenon but an active pathological force. It is fueled by visceral fat and cellular senescence, transmitted via cytokines and EVs, and amplified by gut dysbiosis, which collectively dismantles muscle integrity and destabilizes the vascular wall, thereby inextricably linking the progression of sarcopenia and atherosclerosis (As shown in **Figure 3**).

### 4.3 Ectopic lipid deposition

Ectopic lipid deposition represents a critical physical manifestation of systemic metabolic dysregulation, wherein lipid overflow from dysfunctional adipose tissue infiltrates and compromises non-adipose organs, thereby directly linking the pathogenesis of sarcopenia and atherosclerosis (90, 91). This process, far beyond inert storage, involves the accumulation of bioactive lipid species that actively disrupt cellular signaling and fuel a bidirectional vicious cycle. The initiating event is often the failure of subcutaneous adipose tissue to expand healthily in the face of chronic energy surplus, leading to hypertrophic, hypoxic, and inflamed adipocytes. This dysfunctional state, particularly in visceral fat, results in uncontrolled lipolysis, flooding the circulation with excess FFAs (92) and setting the stage for ectopic deposition (37). The liver and skeletal muscle become primary sinks for this lipid overflow (93).

In skeletal muscle, elevated FFAs are esterified into IMCLs. While IMCLs themselves can be benign energy stores, the specific accumulation of toxic lipid intermediates like DAGs and ceramides is central to pathology (24, 37, 94). DAGs activate novel PKC isoforms, which phosphorylate IRS-1 on serine residues, blunting insulin signaling and contributing to local IR (11, 62, 95). More potently, ceramides activate PP2A and inhibit Akt, the master regulator of anabolism, directly promoting proteolysis and suppressing protein synthesis, thereby driving muscle atrophy (96, 97). This intramyocellular lipotoxicity is now recognized as a key determinant of “muscle quality,” explaining why individuals with similar muscle mass can exhibit vastly different strength and metabolic profiles. Furthermore, lipid droplets can interact with and disrupt mitochondrial membranes, inducing oxidative stress and impairing the energetic capacity necessary for muscle contraction and repair (24).

Concurrently, the liver avidly takes up the excess systemic FFAs, which serve as a substrate for the hepatic overproduction of triglyceride-rich VLDL (98). This VLDL overproduction initiates a cascade of atherogenic lipoprotein remodeling. CETP-mediated exchange transfers triglycerides from VLDL to LDL and HDL in exchange for cholesteryl esters. The resulting triglyceride-enriched LDL and HDL particles become ideal substrates for hepatic lipase, which hydrolyzes the triglycerides, generating sdLDL and small, dense HDL (98). SdLDL particles are highly atherogenic due to their increased susceptibility to oxidation, prolonged circulation half-life, and enhanced propensity for arterial wall retention (49). Meanwhile, the remodeled, dysfunctional HDL loses its capacity to promote reverse cholesterol transport and acquires pro-inflammatory properties, thus failing to protect against atherosclerosis (50, 99).

**Figure 4:**
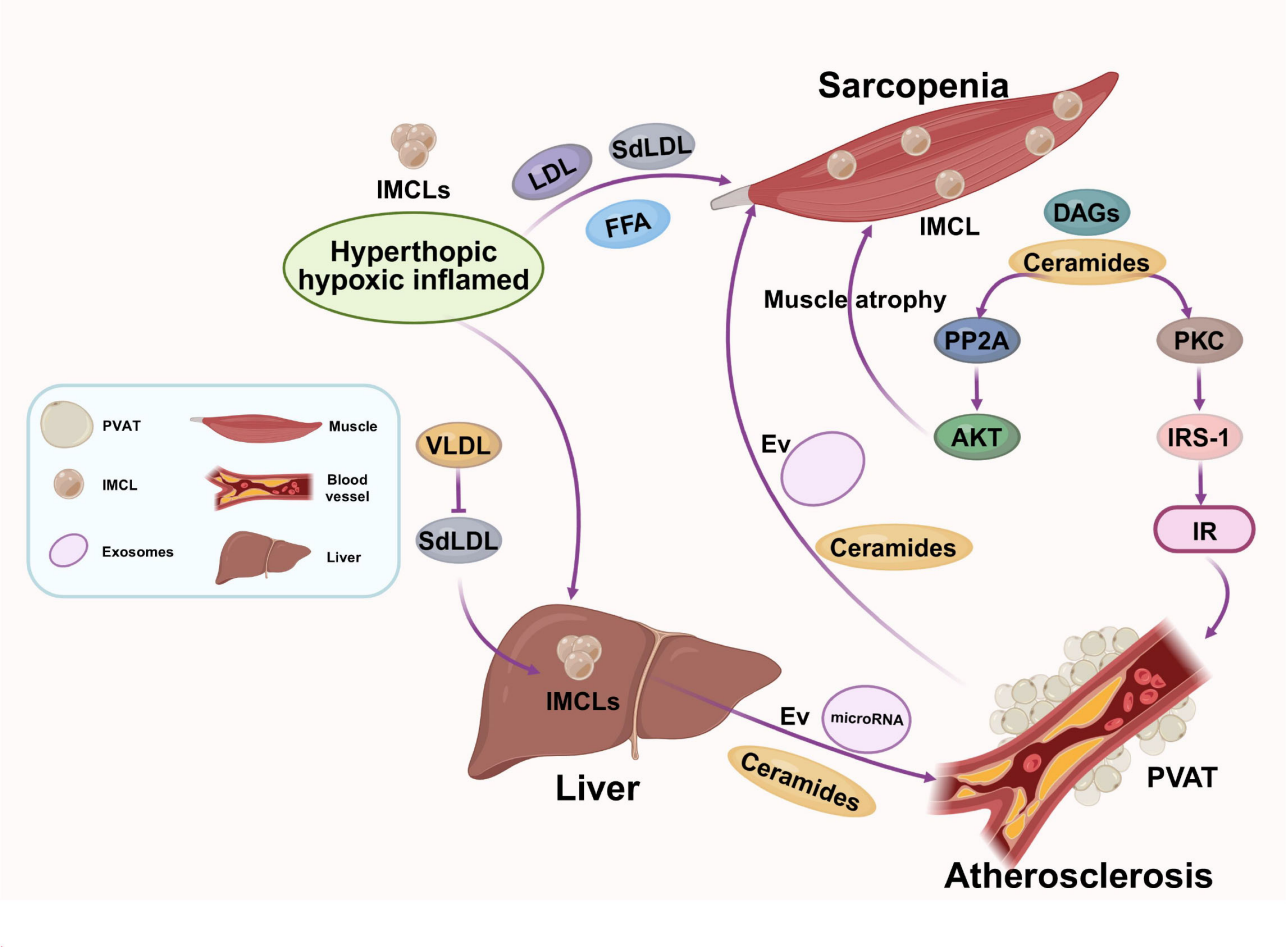
Ectopic lipid deposition drives a shared metabolic pathology between sarcopenia and atherosclerosis. Dysfunctional adipose tissue releases excess FFAs, leading to intramyocellular accumulation of toxic lipids (e.g., ceramides, DAGs) that impair insulin signaling and muscle quality. Concurrently, FFAs drive hepatic overproduction of triglyceride-rich VLDL, which is remodeled into atherogenic sdLDL. This ectopic lipid flux establishes a bidirectional link between muscle lipotoxicity and vascular lipid dysfunction.

Beyond this shared origin in lipid overflow, novel mechanisms underscore the direct crosstalk. The concept of a “muscle-liver-vasculature” axis is gaining traction, where lipotoxins produced in insulin-resistant muscle (e.g., specific ceramide species) can be released into the circulation, potentially influencing hepatic VLDL secretion and directly affecting endothelial function (93, 100). Additionally, EVs derived from steatotic hepatocytes or lipid-laden muscle cells have been shown to carry specific lipid cargo (e.g., ceramides) and microRNAs that can be delivered to recipient cells, such as vascular smooth muscle cells, promoting their phenotypic switch to a pro-calcific, pro-inflammatory state, thereby accelerating atherosclerotic plaque maturation and instability (101, 102). Recent studies also highlight the role of perivascular adipose tissue (PVAT), which, when becoming dysfunctional and lipid-laden, loses its vasoprotective properties and secretes pro-inflammatory adipokines directly onto the adjacent arterial wall, creating a localized inflammatory milieu that accelerates atherosclerosis (38, 103) (As shown in **Figure 4**).

In summary, ectopic lipid deposition is not a passive endpoint but a dynamic and interactive process. It originates from adipose tissue failure, directly impairing muscle function through lipotoxicity, and simultaneously drives atherogenic dyslipidemia. This shared pathway, amplified by emerging inter-organ communication via lipotoxins and EVs, creates a powerful metabolic link that simultaneously deteriorates muscle integrity and vascular health (104, 105).

### 4.4 Hormonal dysregulation

Age-related hormonal alterations create a shared endocrine milieu that predisposes to the parallel progression of sarcopenia and atherosclerosis (106, 107). This phenomenon extends beyond the decline of individual hormones, representing a state of systemic anabolic withdrawal coupled with a pro-inflammatory endocrine shift, which concurrently undermines the maintenance of muscle and vascular integrity.

Vitamin D deficiency, prevalent in aging and cardiometabolic diseases, exerts pleiotropic effects far beyond calcium metabolism. In skeletal muscle, the activation of the nuclear Vitamin D receptor (VDR) is crucial for myogenic differentiation and the maintenance of satellite cell function (108, 109). VDR signaling suppression impairs mitochondrial function and increases expression of atrophy-related genes, leading to sarcopenia (110). In the vasculature, vitamin D deficiency promotes endothelial dysfunction by upregulating the expression of pro-oxidant NADPH oxidase and downregulating eNOS, reducing NO bioavailability (111). Furthermore, it potentiates the Renin-Angiotensin-Aldosterone System (RAAS), leading to increased angiotensin II, which drives vascular inflammation, smooth muscle cell proliferation, and fibrosis, thereby accelerating atherosclerosis (112, 113). Emerging evidence also indicates that vitamin D exerts direct immunomodulatory effects, and its deficiency permits unchecked activation of the NF-κB pathway in both myocytes and vascular cells, amplifying the local inflammatory response (112, 114, 115).

The age-related decline in testosterone and estrogen represents a critical withdrawal of anabolic and vasoprotective support. In skeletal muscle, testosterone directly activates the androgen receptor to stimulate muscle protein synthesis via the Akt/mTOR pathway and inhibits key regulators of proteolysis, such as FoxO1 (116–118). Similarly, estradiol enhances muscle regenerative capacity and attenuates inflammation. Their decline thus creates a net catabolic state. In the vasculature, both hormones are pivotal for endothelial homeostasis. Testosterone and estradiol promote eNOS activation and NO production, ensuring vasodilation and inhibiting endothelial apoptosis. Estradiol, in particular, exerts potent antioxidant effects by suppressing NADPH oxidase and exerts anti-inflammatory actions by inhibiting NF-κB translocation in vascular smooth muscle cells and macrophages (119, 120). The loss of these protective effects post-menopause and in late-onset hypogonadism creates a permissive environment for oxidative stress, inflammation, and the progression of atherosclerotic plaques (121).

The senescence of the GH/IGF-1 axis results in a profound systemic anabolic deficit (122). In muscle, liver-derived and locally paracrine/autocrine IGF-1 binds to the IGF-1 receptor (IGF-1R), activating the canonical PI3K/Akt pathway to promote protein synthesis, inhibit apoptosis, and support satellite cell activity (123–125). Its decline is a central driver of anabolic resistance and muscle wasting. The vascular system is equally dependent on IGF-1 signaling. IGF-1 is a potent survival factor for endothelial cells, stimulating NO production and protecting against oxidative stress-induced apoptosis (126, 127). It also maintains vascular smooth muscle cell contractility and inhibits their pathological transition to a calcifying phenotype. The age-related decline in IGF-1 is thus associated with endothelial dysfunction, increased arterial stiffness, and enhanced susceptibility to vascular calcification (128, 129).

**Figure 5:**
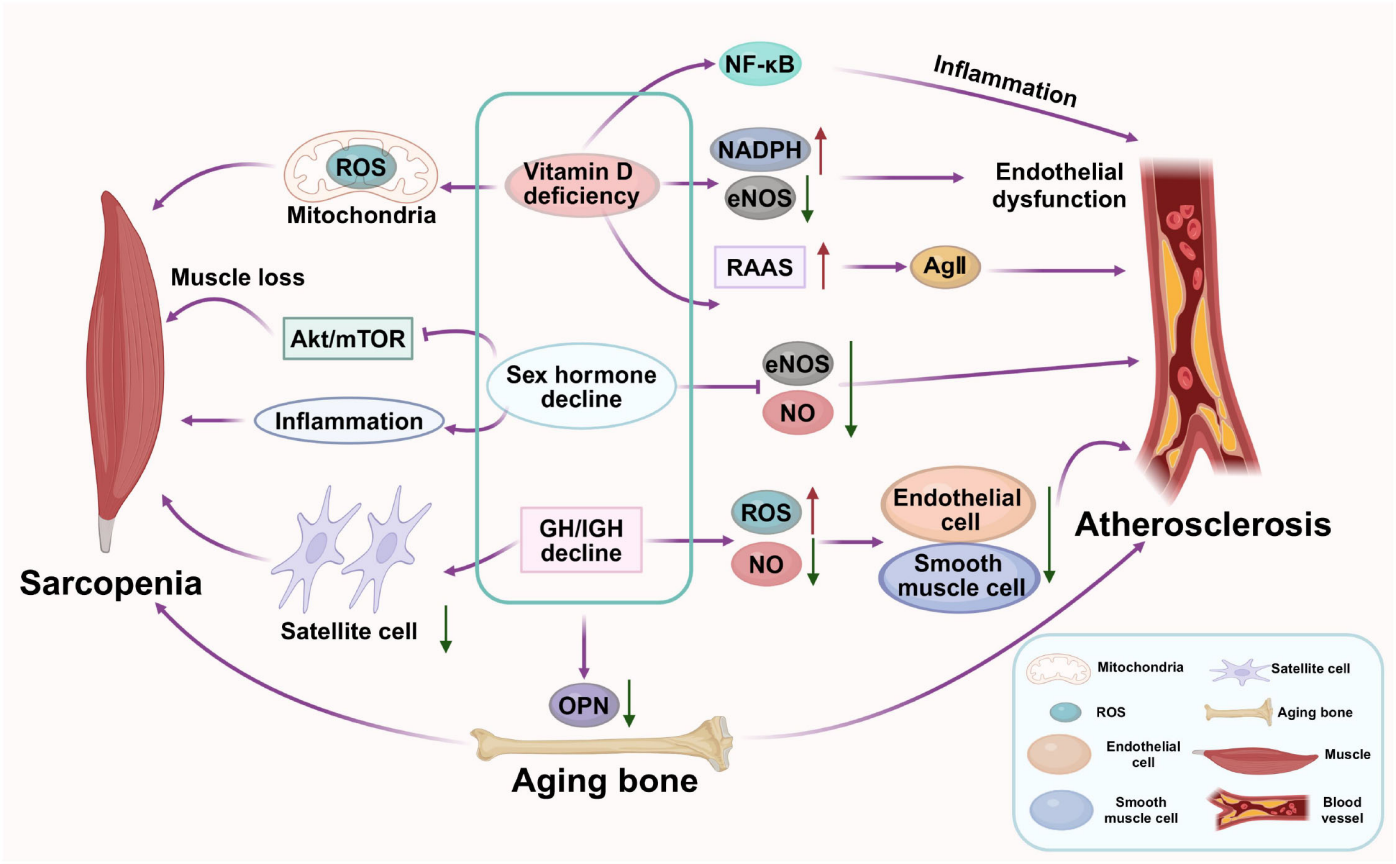
Hormonal dysregulation creates a shared catabolic milieu for sarcopenia and atherosclerosis. Age-related declines in vitamin D, sex hormones, and IGF-1 simultaneously impair muscle anabolism (via suppressed Akt/mTOR signaling) and vascular integrity (via reduced NO bioavailability, increased oxidative stress and inflammation). This shared endocrine deficiency promotes both muscle wasting and endothelial dysfunction, accelerating the co-progression of both conditions.

Beyond these classical axes, recent research highlights the role of bone-muscle cross-talk. Osteocalcin, particularly in its undercarboxylated, hormonally active form, is now recognized to promote muscle function and insulin sensitivity while also exerting protective effects on the endothelium. Age-related decline in osteocalcin may thus represent another endocrine link connecting musculoskeletal decline with vascular aging (130). Similarly, adipose-derived hormones like adiponectin, which typically exerts anti-inflammatory and insulin-sensitizing effects, decline with age and visceral obesity. Low adiponectin levels are associated with both muscle atrophy and accelerated atherosclerosis Thus, the waning of anabolic hormonal support creates a shared environment of vulnerability, predisposing to the parallel progression of muscle wasting and vascular sclerosis (131, 132), highlighting another dimension of the dysregulated endocrine network in aging (131). Moreover, the gut-muscle axis is being elucidated, with evidence suggesting that gut-derived hormones like Ghrelin may not only stimulate appetite but also have direct anti-inflammatory and anabolic effects on skeletal muscle, with potential secondary benefits for vascular health (133) (As shown in **Figure 5**).

In conclusion, hormonal dysregulation in aging creates a catabolic, pro-oxidant, and pro-inflammatory internal environment that simultaneously dismantles the structural and functional integrity of both skeletal muscle and the vascular system (134, 135). This shared endocrine failure provides a powerful rationale for exploring targeted hormone replacement strategies within an integrated gerotherapeutic framework.

## 5 Therapeutic strategies targeting shared metabolic pathways in sarcopenia and atherosclerosis

The recognition of shared pathways necessitates a shift from single-disease management to integrated strategies targeting IR, chronic inflammation, lipid dysregulation, and hormonal shifts to disrupt the bidirectional vicious cycle (4, 136) (As shown in **Table 2**).

**Table 2:** Potential integrated therapeutic strategies targeting shared metabolic pathways in sarcopenia and atherosclerosis.

Therapeutic strategy	Specific intervention	Proposed mechanisms of action	Potential benefits for both conditions
Targeting IR	Combined Exercise (Aerobic + Resistance)	Activates PI3K/Akt/eNOS & AMPK pathways; improves glucose uptake; reduces ectopic fat.	Improved muscle mass/strength; enhanced endothelial function; reduced systemic IR.
	GLP-1 Receptor Agonists	Enhances insulin signaling in muscle and endothelium; reduces ubiquitin ligase expression; anti-inflammatory.	Attenuated muscle atrophy; slowed atherogenesis; improved glycemic control.
	SGLT2 Inhibitors	Promotes glycosuria, ameliorates systemic IR; reduces ectopic lipid deposition.	Improved muscle quality; cardiovascular and renal protection.
Attenuating Inflammaging	Mediterranean/DASH Diet	Modulates gut microbiota; increases SCFAs; reduces pro-inflammatory cytokines (IL-6, TNF-α, CRP).	Reduced systemic inflammation; preserved muscle mass; slowed plaque progression.
	Omega-3 PUFAs (EPA/DHA)	Promotes synthesis of SPMs (e.g., resolvins); competes with pro-inflammatory eicosanoid production.	Mitigation of muscle loss; anti-atherogenic effects; inflammation resolution.
	Senolytics (e.g., Dasatinib + Quercetin)	Clears senescent cells; reduces SASP (IL-6, TNF-α, MMPs).	Improved muscle regeneration and function; enhanced plaque stability.
Correcting Lipid & Ectopic Fat	PCSK9 Inhibitors	Lowers LDL-C; may reduce oxLDL-induced inflammation & intramyocellular lipids.	Improved muscle insulin sensitivity; robust plaque reduction.
	ApoC-III Inhibitors (e.g., Olezarsen)	Reduces triglyceride-rich lipoproteins (VLDL); addresses lipid overflow.	Reduced ectopic fat deposition; lowered CVD risk.
Hormonal Modulation	Vitamin D Supplementation	Modulates NF-κB & RAAS; supports myogenesis and endothelial function.	Improved muscle strength and physical performance; reduced vascular inflammation.
	Testosterone Therapy	Activates Akt/mTOR in muscle; stimulates eNOS in endothelium.	Increased lean mass and strength; improved vascular reactivity.

### 5.1 Targeting IR: the central metabolic defect

Given the pivotal role of IR in both muscle and vascular dysfunction, interventions that enhance insulin sensitivity are foundational.

#### 5.1.1 Pharmacotherapies with dual benefits

GLP-1R agonists (GLP-1RAs) like semaglutide, liraglutide and SGLT2 inhibitors (SGLT2i) like empagliflozin, dapagliflozin, originally developed for T2DM, demonstrate pleiotropic benefits for both muscle and vasculature (137–139). GLP-1 RAs improve skeletal muscle insulin signaling via the Adenosine Monophosphate-Activated Protein Kinase (AMPK)/PI3K pathway, reducing the expression of MuRF-1/Atrogin-1 (7). In parallel, they enhance endothelial function by increasing NO bioavailability and suppressing vascular inflammation (140). SGLT2i, by promoting urinary glucose excretion, ameliorate systemic IR and have been shown to reduce ectopic lipid deposition in muscle and the arterial wall, thereby addressing a key driver of the pathology in both tissues (141).

#### 5.1.2 Exercise as a potent physiological modulator

The integration of aerobic exercise (e.g., brisk walking, cycling) and resistance training (e.g., weight lifting) provides synergistic effects. Aerobic exercise potently activates AMPK, enhancing mitochondrial biogenesis, fatty acid oxidation, and glucose uptake via GLUT4 translocation, thereby ameliorating systemic IR and reducing ectopic lipid deposition (142–146). Concurrently, resistance training directly stimulates the PI3K/Akt/mTOR pathway, promoting muscle protein synthesis and hypertrophy, while also upregulating IRS-1 expression and enhancing insulin sensitivity in muscle (144, 147). Critically, both exercise modalities improve endothelial function through increased shear stress, which upregulates eNOS expression and NO bioavailability, thereby counteracting the endothelial dysfunction central to atherosclerosis (148). Regular exercise also reduces systemic inflammation by lowering circulating levels of pro-inflammatory cytokines (e.g., TNF-α, IL-6) and stimulating the release of anti-inflammatory myokines such as irisin and interleukin-15 from muscle, which promote lipid oxidation and vascular health (149).

In older adults with sarcopenia and cardiovascular disease, combined exercise programs have demonstrated significant improvements in muscle mass, strength, gait speed, and cardiorespiratory fitness, along with reductions in carotid intima-media thickness and arterial stiffness (150, 151). Notably, exercise-induced improvements in muscle quality, are closely correlated with enhanced endothelial function and reduced systemic IR, highlighting the tissue crosstalk facilitated by regular physical activity (152, 153).

### 5.2 Attenuating inflammaging

Systemic low-grade inflammation is a critical connector, driven largely by VAT. Strategies to reduce inflammatory burden are therefore essential.

#### 5.2.1 Anti-inflammatory dietary patterns

The Mediterranean diet (MedDiet), rich in polyphenols, monounsaturated fats, and fiber, exerts potent anti-inflammatory effects by modulating gut microbiota and reducing pro-inflammatory cytokines (e.g., IL-6, TNF-α, CRP) (154). The high fiber content modulates the gut microbiome to promote the production of anti-inflammatory short-chain fatty acids (SCFAs), while its rich profile of polyphenols and monounsaturated fats directly attenuates inflammatory pathways (155). The robust anti-inflammatory and cardioprotective benefits of the MedDiet, which found that supplementation with extra-virgin olive oil or nuts significantly reduced the incidence of cardiovascular events and lowered key inflammatory biomarkers (156, 157), including IL-6, TNF-α, and CRP, reducing the incidence of sarcopenia (158).

Similarly, the Dietary Approaches to Stop Hypertension (DASH) Diet, although originally designed to lower blood pressure, shares relevant anti-inflammatory properties (159). Its emphasis on foods rich in potassium, magnesium, and fiber, coupled with a reduction in saturated fat, confers significant benefits for vascular health. This nutrient profile, by mitigating chronic inflammation, is also highly relevant for preserving muscle health, thereby offering a complementary dietary approach to address the sarcopenia-atherosclerosis comorbidity (160, 161).

#### 5.2.2 Bioactive nutrients and supplements

Long-chain omega-3 polyunsaturated fatty acids (EPA and DHA) attenuate inflammation by competing with arachidonic acid for eicosanoid synthesis and promoting the production of specialized pro-resolving mediators (SPMs) such as resolvins (162, 163). Clinically, supplementation with these fatty acids has been demonstrated to attenuate muscle loss in older adults and slow the progression of atherosclerotic plaques (133, 164, 165).

Adequate high-quality protein intake, particularly leucine-rich sources like whey, stimulates muscle protein synthesis via mTOR activation, countering anabolic resistance (166–168). Furthermore, certain amino acids like arginine support vascular health by serving as a precursor for nitric oxide, a molecule essential for endothelial function (169). Additionally, dietary fiber fermented into SCFAs exerts systemic anti-inflammatory effects via G protein-coupled receptor signaling and Histone Deacetylase inhibition (170–172). Vitamin D supplementation corrects deficiency-related inflammation by modulating NF-κB and RAAS pathways, thereby improving muscle function and endothelial health (114, 173–176). Polyphenols and minerals (magnesium, zinc) further support anti-inflammatory and antioxidant defenses, protecting both muscle and vasculature (177–181).

### 5.3 Correcting lipid dysregulation and ectopic fat deposition

#### 5.3.1 Advanced lipid-lowering agents

PCSK9 inhibitors (e.g., evolocumab) not only reduce LDL-C but also attenuate oxLDL-induced macrophage inflammation and may decrease intramyocellular lipid accumulation, improving muscle quality (182). Novel agents like olezarsen target apolipoprotein C-III to reduce triglyceride-rich lipoproteins and VLDL-C, addressing the lipid overflow that drives ectopic fat deposition (183).

#### 5.3.2 Synthetic biology approaches

Closed-loop gene circuits such as the CHARM system represent an innovative strategy for long-term metabolic regulation. This implantable device senses cholesterol levels and auto-regulates PCSK9 inhibition, normalizing lipid profiles and reducing ectopic fat in preclinical models, offering a potential “set-and-forget” therapeutic platform (182).

### 5.4 Hormonal modulation

Given the role of hormonal decline in both sarcopenia and atherosclerosis, vitamin D and sex hormone replacement therapies hold promise when carefully indicated. Testosterone and estrogen support anabolic signaling via Akt/mTOR in muscle and enhance endothelial NO synthesis in vasculature (116–119, 122, 126, 127). GH/IGF-1 axis modulation may also benefit muscle protein synthesis and vascular repair, though clinical applications require further validation (123–125, 128, 129).

## 6 Conclusion and perspectives

The intricate comorbidity of sarcopenia and atherosclerosis represents a paradigm of multimorbidity rooted in the biology of aging. This review has delineated the bidirectional metabolic crosstalk that fuels a vicious cycle of escalating disability. The clinical imperative is clear: a shift from siloed, disease-specific management toward integrated, mechanism-based interventions that target the shared pillars of aging (21, 26, 37, 38, 47, 183). Emerging evidence underscores the necessity of moving beyond organ-specific approaches toward integrated therapeutic strategies (4). Interventions such as combined exercise training, anti-inflammatory diets (e.g., Mediterranean or DASH diet) (160, 161, 184, 185), and pharmacotherapies with pleiotropic benefits, including GLP-1RAs and SGLT2i (137–139), show promise in simultaneously targeting muscle and vascular health. Additionally, nutritional supplementation with omega-3 fatty acids (162, 163), vitamin D (114, 173–176), and high-quality protein (166–168) may help mitigate anabolic resistance and systemic inflammation (As shown in **Figure 2**).

In conclusion, embracing the geroscience hypothesis, which posits that targeting core aging mechanisms can mitigate multiple age-related diseases, which is paramount for disrupting the vicious cycle linking sarcopenia and atherosclerosis. Future research and clinical translation should be guided by a multi-pronged roadmap targeting fundamental pillars of aging. The clearance of senescent cells via senolytics (e.g., dasatinib/quercetin, fisetin) presents a transformative strategy to attenuate the pro-inflammatory and pro-catabolic SASP that damages both muscle and vasculature, with several clinical trials already underway (2, 3). The integration of multi-omics technologies (proteomics, metabolomics) is unlocking deep phenotyping capabilities, enabling the discovery of novel biomarkers (e.g., specific myokine profiles or gut microbiome-derived metabolites like TMAO) and paving the way for precision geriatrics (55, 186). Concurrently, advanced molecular therapeutics are emerging, including mitophagy inducers (e.g., urolithin A, nicotinamide riboside) to restore mitochondrial quality control (187, 188), gut microbiome engineering to reduce systemic inflammation (189), and innovative RNA therapeutics and gene circuits (e.g., olezarsen, the CHARM system) for long-term management of dyslipidemia (182, 183). Optimizing integrated lifestyle interventions through rigorously defined “doses” of combined exercise and targeted nutrition (e.g., leucine, omega-3s) remains a foundational and potent approach for at-risk older adults (1). Ultimately, by reconceptualizing sarcopenia and atherosclerosis as common downstream outcomes of accelerated organismal aging rather than distinct entities, we can shift the therapeutic paradigm from reactive disease management to proactive targeting of the biological roots of aging, thereby dramatically expanding the health span of our global population.

## Glossary

VDR: Vitamin D receptor
PP2A: Protein Phosphatase 2A
SASP: Senescence-associated secretory phenotype
EVs: Extracellular vesicles
Met-S: metabolic syndrome
T2DM: type 2 diabetes mellitus
CRP: C-reactive protein
IGF-1: Insulin/insulin-like growth factor-1
IGF-1R: IGF-1 receptor
ROS: Reactive Oxygen Species
mtROS: mitochondrial ROS
NADPH: Nicotinamide Adenine Dinucleotide Phosphate
PI3K: Phosphoinositide 3-kinase
IL-1β: Interleukin-1 beta
IL-6: Interleukin-6
TNF-α: Tumor Necrosis Factor-alpha
GH: Growth Hormone
NF-κB: Nuclear Factor-κB
RAAS: Renin-Angiotensin-Aldosterone System
IR: insulin resistance
JAK/STAT: Janus kinase-signal transducer and activator of transcription
OXPHOS: Oxidative phosphorylation
NO: nitric oxide
eNOS: endothelial nitric oxide synthase
PAI-1: Plasminogen activator inhibitor-1
DAGs: Diacylglycerols
VCAM-1: Vascular Cell Adhesion Molecule-1
ICAM-1: Intercellular Adhesion Molecule-1
AKT/mTOR: Protein Kinase B/Mammalian Target of Rapamycin
ET-1: endothelin-1
VLDL: very-low-density lipoproteins
oxLDL: oxidized low-density lipoproteins
GLP-1: Glucagon-Like Peptide-1
SGLT2: sodium-dependent glucose transporter 2
AMPK: Adenosine Monophosphate - Activated Protein Kinase
CETP: cholesteryl ester transfer protein
MMPs: matrix metalloproteinases
VAT: visceral adipose tissue
PVAT: perivascular adipose tissue
IMCL: Intramyocellular Lipids
TMAO: trimethylamine N-oxide
MedDiet: Mediterranean diet
DASH: Dietary Approaches to Stop Hypertension
FoxO: Forkhead Box O
IL-6R: interleukin-6 receptor
sdLDL: Small-dense LDL
SCFAs: short-chain fatty acids
SPMs: Specialized pro-resolving mediators
